# Supplementary material for: Identification of photoperiod-induced specific miRNAs in the adrenal glands of Sunite sheep (Ovis aries)
Source: Front Vet Sci. 2022 Jul 22;9:888207. doi: 10.3389/fvets.2022.888207 (PMC9354845; doi:10.3389/fvets.2022.888207)
Supplement: Supplementary file 1 [file Data_Sheet_1.ZIP › Supplementary file/miRDeep2 file.zip!/miRDeep2 file/Novel_Structure/SP42A2/Novel_224.pdf]

| | | |
|---|---|---|
| Provisional ID | : | NC_019465.2_18811 |
| Score total | : | 1 |
| Score for star read(s) | : | -1.3 |
| Score for read counts | : | 0 |
| Score for mfe | : | 0.7 |
| Score for randfold | : | 1.6 |
| Score for cons. seed | : | |
| Total read count | : | 25 |
| Mature read count | : | 24 |
| Loop read count | : | 0 |
| Star read count | : | 1 |

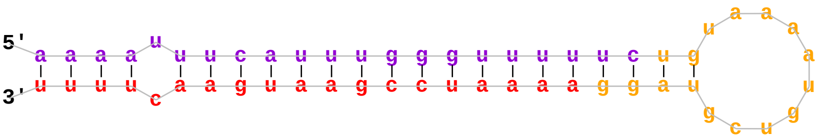


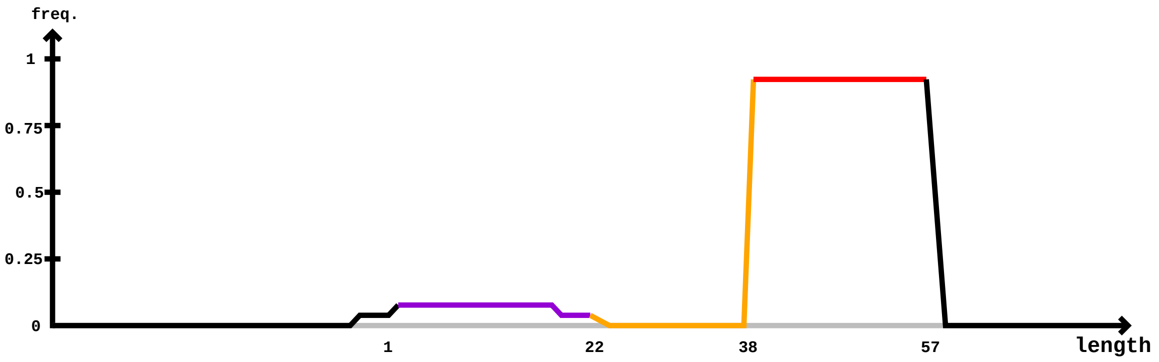


**Star** **Mature**

```
5'- ucuuuaaggauccauguaauuauugccugggucaaaaauuucauuuggguuuuucuguaaaaugucguaggaaaauccgaaugaacuuuucuggucaaaccaauagcuga  -3'   obs
    ucuuuaaggauccauguaauuauugccugggucaaaaauuucauuuggguuuuucuguaaaaugucguaggaaaauccgaaugaacuuuucuggucaaaccaauagcuga        exp
    ........((((((.((((...)))).)))))).((((.((((((((((((((((((..........)))))))))))))))))).)))).((((...))))........  reads   mm   sample
    ..............................gucaaaaaAuucauuuggguu...........................................................  1       1    seq
    ..................................aaaaGuucauuuggguuuuuc.......................................................  1       1    seq
    .......................................................................aaaaucUgaaugaacuuuu....................  23      1    seq
    .......................................................................aaaauccgaaugaacuuuuc...................  1       0    seq
```
